# High efficiency classification of children with autism spectrum disorder

**DOI:** 10.1371/journal.pone.0192867

**Published:** 2018-02-15

**Authors:** Genyuan Li, Olivia Lee, Herschel Rabitz

**Affiliations:** 1 Department of Chemistry, Princeton University, Princeton, New Jersey 08544, United States of America; 2 Peddie School, Hightstown, New Jersey 08520, United States of America; Indraprastha Institute of Information Technology, INDIA

## Abstract

Autism spectrum disorder (ASD) is a wide-ranging collection of developmental diseases with varying symptoms and degrees of disability. Currently, ASD is diagnosed mainly with psychometric tools, often unable to provide an early and reliable diagnosis. Recently, biochemical methods are being explored as a means to meet the latter need. For example, an increased predisposition to ASD has been associated with abnormalities of metabolites in folate-dependent one carbon metabolism (FOCM) and transsulfuration (TS). Multiple metabolites in the FOCM/TS pathways have been measured, and statistical analysis tools employed to identify certain metabolites that are closely related to ASD. The prime difficulty in such biochemical studies comes from (i) inefficient determination of *which* metabolites are most important and (ii) understanding *how* these metabolites are collectively related to ASD. This paper presents a new method based on scores produced in Support Vector Machine (SVM) modeling combined with High Dimensional Model Representation (HDMR) sensitivity analysis. The new method effectively and efficiently identifies the key causative metabolites in FOCM/TS pathways, ranks their importance, and discovers their independent and correlative action patterns upon ASD. Such information is valuable not only for providing a foundation for a pathological interpretation but also for potentially providing an early, reliable diagnosis ideally leading to a subsequent comprehensive treatment of ASD. With only tens of SVM model runs, the new method can identify the combinations of the most important metabolites in the FOCM/TS pathways that lead to ASD. Previous efforts to find these metabolites required hundreds of thousands of model runs with the same data.

## Introduction

Autism Spectrum Disorder (ASD) is a serious developmental disease that is characterized by difficulty in socializing, communicating, and interacting with others. According to research done by the Center for Disease Control and Prevention, in 2000, an average of 1 in every 150, while in 2012 about 1 in every 68 (∼1.5%) American children were diagnosed with autism [1]. Some symptoms of ASD are not evident until age two or later. In other cases, a child may appear to be developing normally until age two, and then may stop learning new skills, or may even forget old skills [2]. Psychometric tools are often used to diagnose ASD. The Childhood Autism Rating Scale (CARS) [3] and Autism Diagnostic Observation Scale (ADOS) [4] are two instruments used for ASD diagnosis, which utilizes behavioral observations from parents, teachers, and caregivers. However, “in the current practice, diagnosis often has to be delayed until the behavioral symptoms become evident during childhood” [5], which prevents the child from getting prompt treatment.

ASD is not necessarily just a genetic disorder, as it is thought that environmental effects also contribute to ASD’s etiology [6]. Recently, researchers have been developing a biochemical approach that is able to diagnose a significant portion of ASD cases. There have been observed differences in FOCM/TS pathways between children with ASD and individuals considered as disease free neurotypical controls (NEU) [7].

Multivariate statistical analysis was used by Howsmon et al. [8] to obtain information utilized to distinguish ASD from NEU, and to draw correlations between metabolite measurements and the severity of ASD. In particular, twenty four measurements of the metabolites in FOCM/TS pathways listed in Table 1 were utilized. A linear classifier based on Fisher Discriminant Analysis (FDA) [9, 10] was then used to distinguish ASD and NEU participants. The cross-validated misclassification rates were only 4.9% and 3.4% for the NEU (76 normal children) and ASD (83 patients) samples, respectively.

**Table 1:** FOCM/TS metabolites considered for analysis [8].

Variable	Metabolite	Variable	Metabolite
*x*_1_	Methionine	*x*_13_	fGSH
*x*_2_	SAM	*x*_14_	GSSG
*x*_3_	SAH	*x*_15_	fGSH/GSSG
*x*_4_	SAM/SAH	*x*_16_	tGSH/GSSG
*x*_5_	% DNA methylation	*x*_17_	Chlorotyrosine
*x*_6_	8-OHG	*x*_18_	Nitrotyrosine
*x*_7_	Adenosine	*x*_19_	Tyrosine
*x*_8_	Homocysteine	*x*_20_	Tryptophan
*x*_9_	Cysteine	*x*_21_	fCystine
*x*_10_	Glu.-Cys.	*x*_22_	fCysteine
*x*_11_	Cys.-Gly.	*x*_23_	fCystine/fCysteine
*x*_12_	tGSH	*x*_24_	% oxidized glutathione

Not all the metabolites listed in Table 1 are necessary for classification of ASD and NEU. Using all of them may not only lead to overfitting, but also fail to distinguish the key causative and less-informative metabolites. To avoid overfitting caused by simultaneous use of multiple metabolites, Howsmon et al. used cross-validation. They further mitigated over-fitting problems by selecting only a minimum number of metabolites required to adequately classify the ASD and NEU groups [8]. The *wrapper* method [11] was used to *evaluate the performance of the chosen learning algorithm (i.e., FDA) for all possible combinations of up to six metabolites* for FDA classification to find the best combination. Then they selected combinations of higher numbers of metabolites in a greedy fashion to sequentially add additional metabolites that best improved the classification of the original identified best six metabolites. The selected best combination of seven metabolites given in the text and the caption of Fig 5 in Howsmon’s paper [8] are given in Table 2.

**Table 2:** The best combination of metabolites selected by wrapper method [8].

Variable	Metabolite
*x*_5_	% DNA methylation
*x*_6_	8-OHG
*x*_10_	Glu.-Cys.
*x*_23_	fCystine/fCysteine
*x*_24_	% oxidized glutathione
*x*_17_	Chlorotyrosine
*x*_16_	tGSH/GSSG

The wrapper method has three shortcomings: 1) there are

$$\begin{array}{c} \sum_{i=1}^{6}C_{24}^{i}=190,050 \end{array}$$

combinations of up to 6 members selected from the 24 metabolites, and evaluation of all these combinations requires running FDA 190,050 times, which is very computationally demanding. If the number of variables (metabolites here) is large (e.g., hundreds or thousands often occur in various biochemical data sets), testing for all possible combinations is infeasible; 2) the wrapper method provides no information about which members of the identified metabolites contribute the most in classification of ASD; and 3) the procedure does not reveal the relationship between the identified metabolites, i.e., whether they contribute independently or correlatively.

The problems posed above are a general challenge in machine learning: *feature* (metabolite here) *selection, prioritization and correlation identification*. Note that in the remainder of the paper, we will interchangeably use the words: *metabolites, features, or (input) variables* to avoid confusion or maintain terminology that is standardly used with various algorithms in the paper.

Feature selection is the process of finding a small subset of significant variables that have good classification performance. Feature selection algorithms may be conveniently grouped into two categories: *filter* and *wrapper* methods. In contrast to wrapper methods, filter methods rely on the application of an *univariate criterion* to each feature separately in order to select the important feature subsets without running the chosen learning algorithm [11].

For example, the *t*-test is a filter method most commonly used for feature selection and prioritization [12]. In this case, the data are separated into two sets according to their grouping, e.g., “NEU” and “ASD”. Then, a comparison of the two data sets for each feature, *x*_*i*_, by *t*-test is performed under the null hypothesis that the two data sets for *x*_*i*_ are drawn from the same normal distribution. The *p*-value obtained in the *t*-test for each *x*_*i*_ is used as an univariate criterion of how effective *x*_*i*_ is at separating groups. The larger the *p*-value for *x*_*i*_, the less effective *x*_*i*_ is. In this fashion, the magnitudes of the *p*-value for all features inversely define their prioritization order. An appropriate threshold for the *p*-value needs to be set for feature selection. If the *p*-value is close to zero (e.g., less than the threshold 0.05 or 0.01), the null hypothesis is rejected, i.e., the two data sets of *x*_*i*_ may not come from the same distribution, and *x*_*i*_ is most probably a causative feature. Otherwise, *x*_*i*_ is not a causative feature and can be removed. However, for experimental and clinical data, the assumption of a normal distribution may not be valid. Furthermore, if the sample size is small, the comparison of two data set distributions is often not reliable; in this case the *t*-test may not give a correct answer.

Compared to wrapper methods, filter methods are simple and fast as they do not need to run a learning algorithm. Moreover, filter methods treat each feature separately, the number of features does not have an influence on its performance, and thus filter methods can handle very high dimensional systems.

In this paper we propose a new *two stage* method based on two univariate criteria: (i) the sensitivity index, *main effect* ${\hat{S}}_{i}\;\left(i=1,2,...,n\right)$, defined by the variance-based method [13–16], and (ii) the sensitivity *structural* (*independent*) *index* $S_{i}^{a}\;\left(i=1,2,...,n\right)$, deduced from Structural and Correlative Sensitivity Analysis (SCSA) based on HDMR [17, 18]. This dual analysis method can treat data of *small size and with an arbitrary probability distribution*. The proposed method is a special filter method based on the Support Vector Machine (SVM) learning algorithm. For illustration of the proposed method, the same ASD data used by Howsmon et al. [8] will be treated by this new two stage method.

Both ${\hat{S}}_{i}$ and $S_{i}^{a}$ are positive quantities. The larger the value of ${\hat{S}}_{i}$ or $S_{i}^{a}$, then *x*_*i*_ is concluded to be more important. Thus, the magnitudes of ${\hat{S}}_{i}$ or $S_{i}^{a}$ lead to a prioritization order for input variables. Moreover, the additional *correlative* sensitivity index $S_{i}^{b}$ from SCSA discovers the correlative action patterns of the identified metabolites upon ASD. With only tens of SVM model runs, the new method identifies the combinations of the most important metabolites in the FOCM/TS pathways that lead to ASD. In contrast, to find these metabolites, Howsmon et al. performed hundreds of thousands of FDA model runs. Furthermore, the information about the importance order and the correlative action patterns of the identified metabolites for ASD predisposition provides valuable additional insight for a deeper understanding of ASD mechanism and a possible future path to its treatment. The newly introduced analysis tools are general and should be applicable for other diseases requiring a like analysis to reveal their biological origins.

## Methods

To understand the two stage method for feature selection, prioritization and correlation identification, we need some knowledge of HDMR sensitivity analysis.

**Fig 1:**
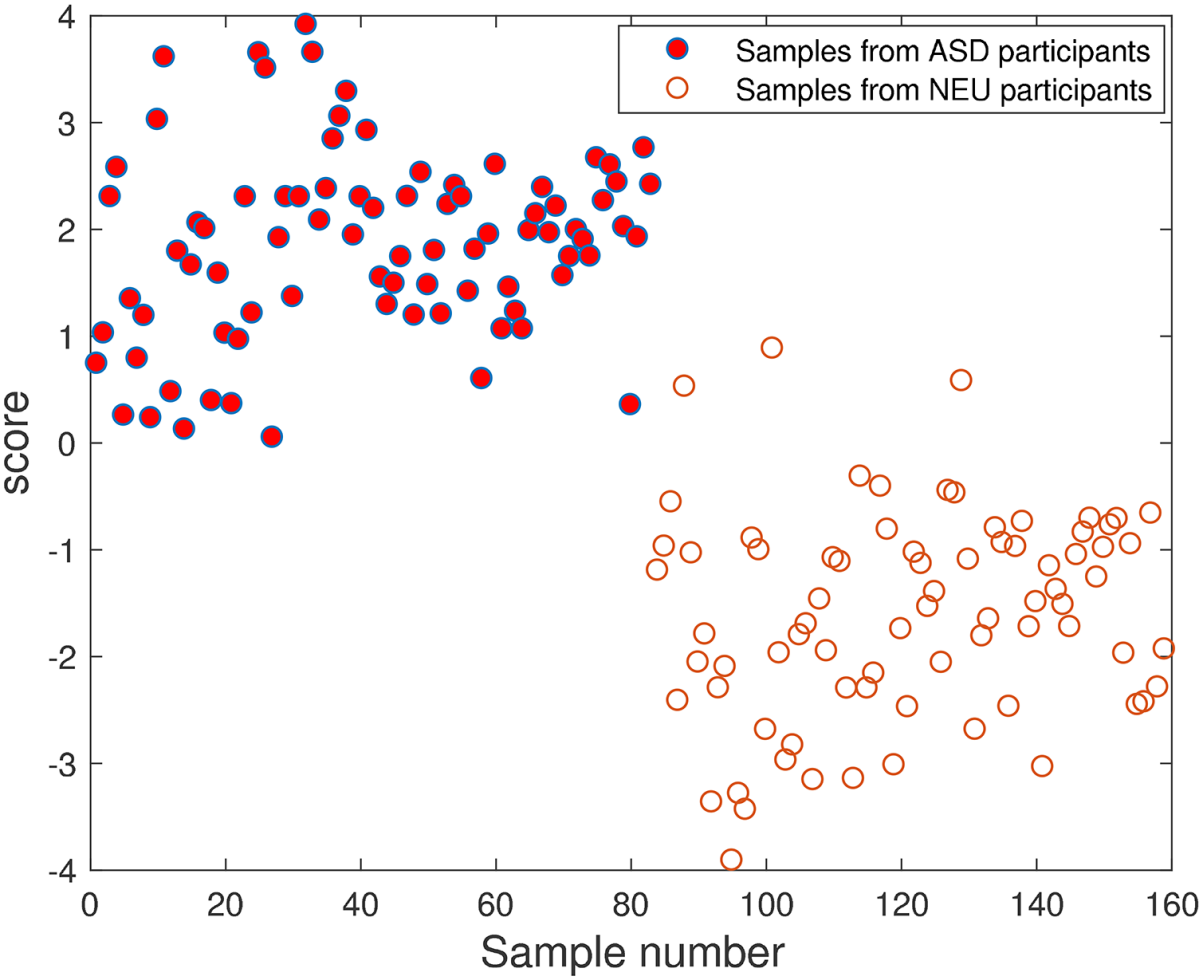
The score of the SVM model for the ASD-NEU data with all the 24 metabolites as input variables (ASD is set to be 1, and NEU is set to be −1 in SVM classification).

Here the principles of HDMR sensitivity analysis are briefly summarized. The details can be found in references [17–20]. We will consider sensitivity indexes of a continuous output *y* with respect to the input variables **x** = (*x*_1_, *x*_2_, …, *x*_*n*_)^*T*^

$$\begin{array}{c} y=f(x), \end{array}$$ (1)

where *f* is the function for **x** → *y*. In classification, the output *y* is a categorical variable representing labels, like [NEU, ASD] or equivalently [−1, 1]. In this case, sensitivity analysis utilizing proposed two indexes, ${\hat{S}}_{i}$ and $S_{i}^{a}$, cannot be readily performed. Fortunately, in many classification learning algorithms (e.g., FDA and SVM), the *implicit* output *y* actually is a continuous variable referred to as *score*. Fig 1 gives the output score of the SVM model with a linear kernel for the ASD-NEU data using all the 24 metabolites as input variables **x**.

The *explicit* output of SVM is given by the sign function of the score, i.e.,

$$\begin{array}{c} \text{sgn}\left(y\right)≔\{\begin{array}{cc} -1 & \text{if}\;\;y<0,\\ 0 & \text{if}\;\;y=0,\\ 1 & \text{if}\;\;y>0. \end{array} \end{array}$$ (2)

**Fig 2:**
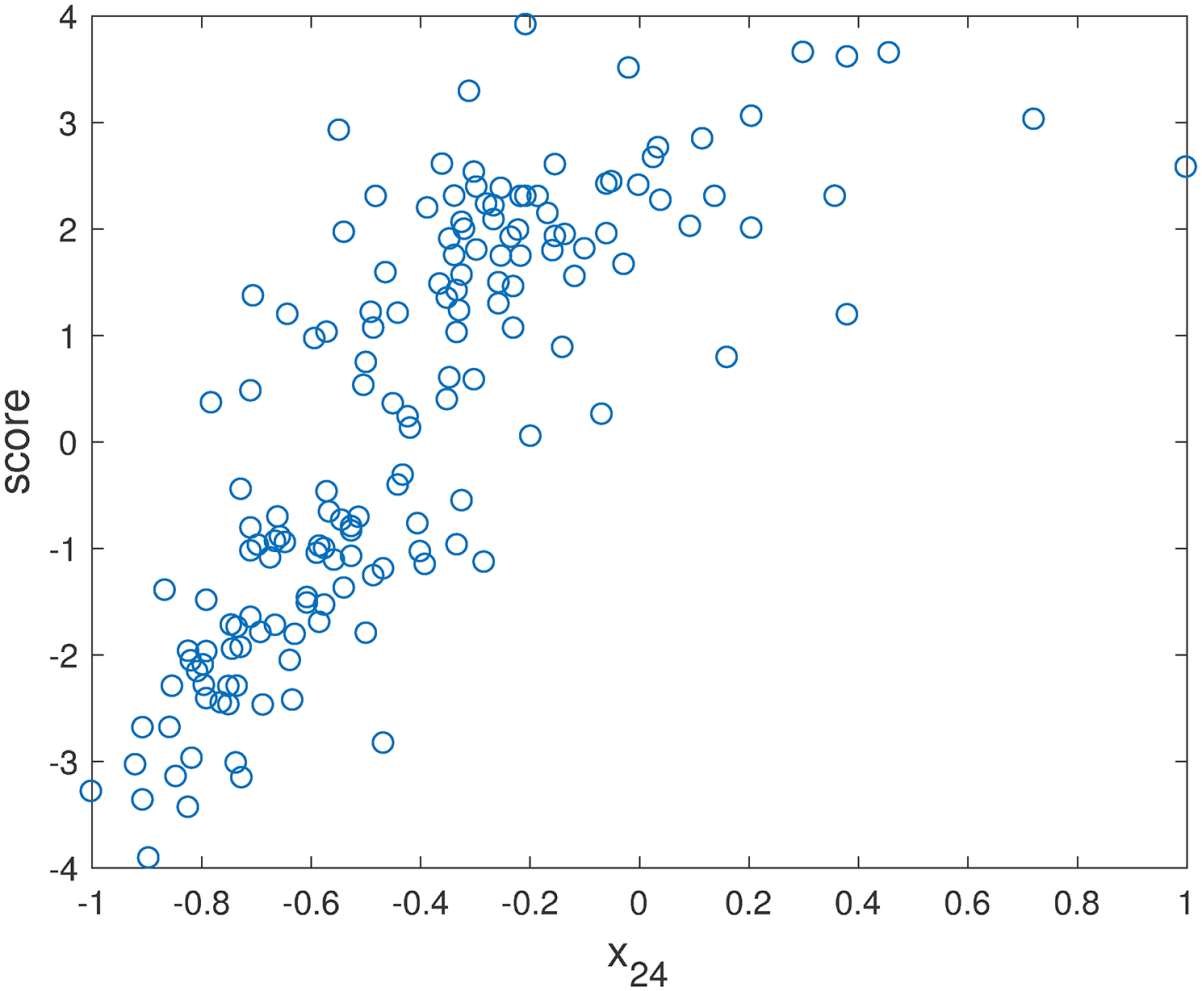
Relation between the score of the SVM model with 24 input variables versus the particular input variable *x*_24_ (% oxidized glutathione).

Using the SVM classification score as the continuous output, then the classification problem may be treated by regression (Support Vector Regression (SVR)) [21], and sensitivity analysis can be readily performed. Fig 2 plots the relation between the score of the SVM model with all 24 metabolites as input variables **x** versus variable *x*_24_ (% oxidized glutathione) in ASD-NEU data.

Following this procedure we may identify *the most important feature as the one whose variation has the largest influence on the variation of the output score*.

### Sensitivity index: Main effect ${\hat{S}}_{i}$

The sensitivity index, main effect ${\hat{S}}_{i}$ is a commonly used measure for ranking the importance of input variables, defined by the variance-based method as [13–16]

$$\begin{array}{c} {\hat{S}}_{i}=\frac{V_{x_{i}}\left[E_{x_{-i}}\left(f\left(x|x_{i}\right)\right)\right]}{V(f(x))},\;\;\;i\in \left\{1,2,...,n\right\}. \end{array}$$ (3)

where $V_{x_{i}}$ and $E_{x_{-i}}$ denote the conditional variance and conditional expectation operators with respect to *x*_*i*_ and **x**_−*i*_ = (*x*_1_, *x*_2_, …, *x*_*i*−1_, *x*_*i*+1_, …, *x*_*n*_)^*T*^, respectively, and V(f(x)) denotes the unconditional variance of the output. The output *y* = *f*(**x**) is a continuous variable given by the function *f*(**x**). ${\hat{S}}_{i}$ reflects *the portion of the output variance caused by the variation of the input variable x_i_*. The larger the value of ${\hat{S}}_{i}$, then the more *x*_*i*_ contributes to the variance of the output. Thus, ${\hat{S}}_{i}$ is a well established univariate criterion to rank the importance of input variables and can be used for feature selection and prioritization.

The determination of ${\hat{S}}_{i}$ by the traditional variance-based methods above is quite computationally demanding and requires a large number (thousands or more) of specifically designed samples based on *assumed knowledge* of the probability distribution of the input variables [14–16, 22, 23]. However, for experimental and clinical data, the input probability distributions are often explicitly unknown. Therefore, traditional variance-based methods cannot be used to treat the latter types of data. A new algorithm to estimate ${\hat{S}}_{i}$ from a limited number of experimental or clinical samples has been developed without requiring explicit knowledge of the probability distribution of the input variables [19].

**Fig 3:**
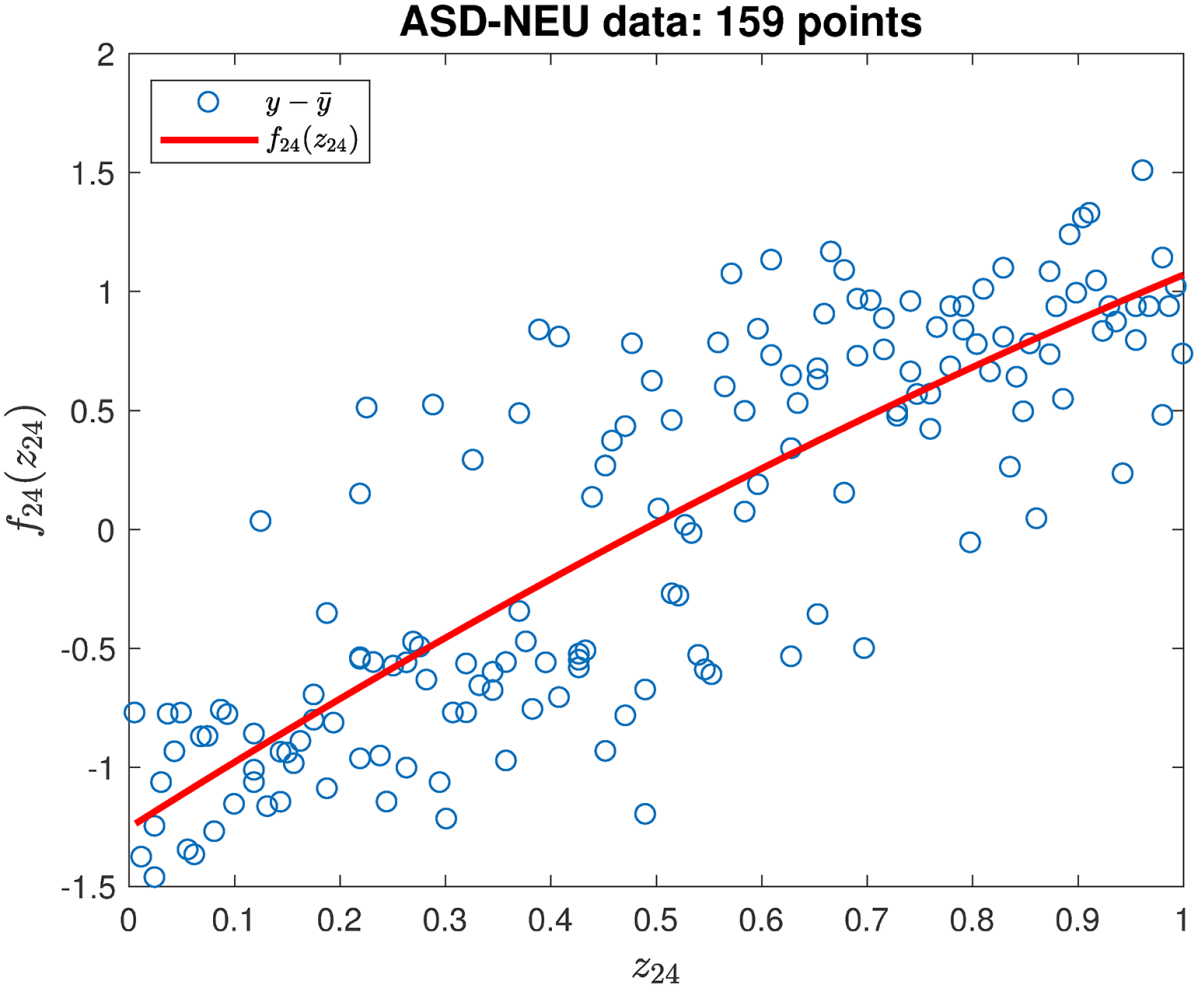
Least squares regression for *f*_24_(*z*_24_) with respect to *z*_24_.

First, the variable *x*_*i*_ is transformed to a new independent variable *z*_*i*_ uniformly distributed in [0, 1] by the Rosenblatt transformation [24]

$$\begin{array}{c} z_{i}=P\left\{X_{i}\leq x_{i}\right\}=F_{i}\left(x_{i}\right), \end{array}$$ (4)

where *F* denotes the cumulative distribution function (cdf). Many numerical methods have been developed for empirical determination of a cdf from the data [25–27]. Matlab has a code *ecdf* for this purpose. As *z*_*i*_ is an independent variable, the first order HDMR component function *f*_*i*_(*z*_*i*_) for *z*_*i*_ can then be determined as [19]

$$\begin{array}{c} f_{i}\left(z_{i}\right)=\int_{{\left[0,1\right]}^{n-1}}f\left(z_{i},x_{-i}\right)dx_{-i}-\bar{y}, \end{array}$$ (5)

where $\bar{y}$ is the mean value of the output *y* for all samples. This procedure is equivalent to determining *f*_*i*_(*z*_*i*_) by least squares regression from *z*_*i*_ and all outputs *f*(*z*_*i*_, **x**_−*i*_) at the same value of *z*_*i*_. Fig 3 gives the least squares regression for *f*_24_(*z*_24_) with respect to *z*_24_ in the ASD-NEU data.

As *z*_*i*_ is a function of *x*_*i*_ only, the main effect, ${\hat{S}}_{i}$, for *x*_*i*_ can be estimated as [19]

$$\begin{array}{c} {\hat{S}}_{i}\approx \frac{\frac{1}{N}\sum_{s=1}^{N}f_{i}^{2}\left(z_{i}^{\left(s\right)}\right)}{\frac{1}{N}\sum_{s=1}^{N}{\left(y^{\left(s\right)}-\bar{y}\right)}^{2}},\;i\in \left\{1,2,...,n\right\} \end{array}$$ (6)

where $z_{i}^{\left(s\right)}$ and *y*^(*s*)^ are the values of *z*_*i*_ and *y* in the *s*th sample; *N* is the total number of samples used to determine ${\hat{S}}_{i}$.

Since ${\hat{S}}_{i}$ is determined separately for each *x*_*i*_, the number of input variables does not have an influence on its determination. Thus, ${\hat{S}}_{i}$ can be used to treat vary high (e.g., thousands and more) dimensional systems. Still a shortcoming of using ${\hat{S}}_{i}$ is that it contains the contributions from other *x*_*j*_’s correlated to *x*_*i*_ [19]. When the correlation is positive (negative), ${\hat{S}}_{i}$ is larger (smaller) than the independent contribution of *x*_*i*_. The resulting ordering of the features from ${\hat{S}}_{i}$ then may incorrectly represent the independent contributions of features. Therefore, ${\hat{S}}_{i}$ will be used *first* in the two stage method for feature *pre-selection* to remove the *most insignificant* input variables which is especially important to perform when the number of input variables is large.

### Determination of SCSA indexes

SCSA is based on HDMR with independent and/or correlated input variables [17]. A newly developed svr-based HDMR algorithm with *independent* input variables and *known* probability distributions of inputs is efficient for HDMR modeling and sensitivity analysis with a modest number of samples [20]. As the variables in experimental and clinical data are often correlated and their probability distributions are explicitly unknown, here, we *extend* the above svr-based HDMR algorithm to correlated variables. As shown below, without the knowledge of the variable probability distribution the first order HDMR expansion with correlated variables still can be constructed from experimental and clinical data, and will be used to determine the first order SCSA indexes for ASD-NEU data.

#### HDMR and SCSA

Many problems in science and engineering reduce to the need for efficiently and functionally describing the relationship between a set of high dimensional system input variables **x** = (*x*_1_, …, *x*_*n*_)^*T*^ and the system output *y* = *f*(**x**). As the contributions of the multiple input variables *upon* the output can act *individually* and *interactively*, it is natural to express the (explicitly known or unknown) function *f*(**x**) as a finite hierarchical expansion [18]:

$$\begin{array}{ccc} f(x) & = & f_{0}+\sum_{i=1}^{n}f_{i}\left(x_{i}\right)+\sum_{1\leq i<j\leq n}f_{ij}\left(x_{i},x_{j}\right)\\ & + & ...+f_{12...n}\left(x_{1},x_{2},...,x_{n}\right)\\ & = & \sum_{u\subseteq \{1,2,...,n\}}f_{u}\left(x_{u}\right). \end{array}$$ (7)

where *u* is a subset in {1, 2, …, *n*} including the empty set ∅, (i.e., *f*_∅_(**x**_∅_) = *f*_0_) and **x**_*u*_ are the elements of **x** whose indexes are in *u*. For simplicity, in sequel we will write *u* ⊆ *n* in place of *u* ⊆ {1, 2, …, *n*}. When the component functions satisfy the *hierarchical orthogonality* condition (i.e., they are optimally defined to maximize the contribution of low order component functions), the above expansion is referred to as an HDMR expansion. For many systems, the higher order HDMR component functions are negligible, and *f*(**x**) can be approximated by a low (e.g., first or second) order HDMR expansion. For the ASD-NEU metabolite system in this paper, the first order HDMR expansion

$$\begin{array}{c} f\left(x\right)=f_{0}+\sum_{i=1}^{n}f_{i}\left(x_{i}\right) \end{array}$$ (8)

was found to give a very good approximation, where *f*_0_ is a constant representing the mean contribution of all input variables, and *f*_*i*_(*x*_*i*_) represents the contribution of *x*_*i*_ to the output.

Based on a covariance decomposition of the unconditional variance of the output, a general global sensitivity analysis for independent and correlated variables, referred as structural (independent) and correlative sensitivity analysis (SCSA) was proposed [17, 18].

V(f(x))=E[(f(x)-f0)2]=E[∑∅≠u⊆nfu(xu)(f(x)-f0)]=∑∅≠u⊆n[V(fu(xu))+Cov(fu(xu),∑∅≠v⊆nu≠vfv(xv))], (9)

where Cov(⋅) denotes covariance, and the property of zero expectation $E(f_{u}\left(x_{u}\right))=0$ for the HDMR component functions was used. The SCSA sensitivity indexes are defined by normalization, i.e., by dividing both sides of Eq (9) with V(f(x)).

1=∑∅≠u⊆n[V(fu(xu))V(f(x))+Cov(fu(xu),∑∅≠v⊆nu≠vfv(xv))V(f(x))]=∑∅≠u⊆n[Sua+Sub]=∑∅≠u⊆nSu. (10)

Here, for **x**_*u*_ we denote $S_{u}^{a}$ as the structural (independent) contribution (i.e., related to *f*_*u*_(**x**_*u*_) and the marginal probability density function (pdf), *p*_*u*_(**x**_*u*_), only), $S_{u}^{b}$ as the correlative contribution (i.e., related to *f*_*u*_(**x**_*u*_), other component functions *f*_*v*_(**x**_*v*_)’s and the joint pdf, *p*(**x**)) and *S*_*u*_ as the total contribution equal to

$$\begin{array}{c} S_{u}=S_{u}^{a}+S_{u}^{b}. \end{array}$$ (11)

Especially, for *u* = {*i*}, we have

$$\begin{array}{c} S_{i}=S_{i}^{a}+S_{i}^{b}, \end{array}$$ (12)

the first order SCSA indexes for variable *x*_*i*_.

We can also consider the correlation of each pair of variables by computing

$$\begin{array}{ccc} S^{b}\left(ij\right) & = & \frac{\text{Cov}(f_{i}\left(x_{i}\right),f_{j}\left(x_{j}\right))}{V(f(x))}\\ & \approx & \frac{\frac{1}{N}\sum_{s=1}^{N}f_{i}\left(x_{i}^{\left(s\right)}\right)f_{j}\left(x_{j}^{\left(s\right)}\right)}{\frac{1}{N}\sum_{s=1}^{N}{\left(y^{\left(s\right)}-\bar{y}\right)}^{2}},\;i,j\in \left\{1,2,...,n\right\}. \end{array}$$ (13)

Note that

$$\begin{array}{c} S^{b}\left(i,i\right)=S_{i}^{a},\;\sum_{j=1,j\neq i}^{n}S^{b}\left(ij\right)=S_{i}^{b},\;\sum_{j=1}^{n}S^{b}\left(ij\right)=S_{i}. \end{array}$$ (14)

SCSA *separates* the independent and correlative contributions of the input variable *x*_*i*_. In particular, $S_{i}^{a}$ is referred to as the *structural* index giving the independent contribution of input variable *x*_*i*_ upon the variation of *y*, while the $S_{i}^{b}$ index gives the *correlative* contribution of *x*_*i*_ arising from all other variables, *x*_*j*_’s, correlated with *x*_*i*_. Hence, $S_{i}^{a}$ is an *ideal* univariate criterion for feature selection and prioritization, and $S_{i}^{b}$ is used for correlation identification. *S*^*b*^(*ij*) can be considered as a *nonlinear correlation coefficient* for variables *x*_*i*_ and *x*_*j*_. *S*_*i*_ is referred to as the *total* index. When the output is *satisfactorily approximated* by the first order HDMR expansion, the sum of all total indexes should satisfy [18]

$$\begin{array}{c} \sum_{i=1}^{n}S_{i}\approx 1. \end{array}$$ (15)

The closer ∑_*i*_ *S*_*i*_ is to 1, then the first order sensitivity analysis is deemed more reliable. Furthermore, the first order HDMR component *f*_*i*_(*x*_*i*_) as a function of *x*_*i*_ provides the influence pattern of *x*_*i*_ upon the output *y*.

The advantage of the first order SCSA indexes, $S_{i}^{a},S_{i}^{b},S_{i}$, lies in their ability to perform feature selection, prioritization and correlation identification to good accuracy even with small size data samples (e.g., in the present case, there are 169 points of ASD-NEU data). SCSA requires the construction of an HDMR model utilizing all input variables, and thus it is more reliable than ${\hat{S}}_{i}$ or the *t*-test which only employ information for each input variable separately. This situation becomes significant when the sample size is small.

A shortcoming of performing SCSA is that it is difficult to treat very high dimensional systems because construction of an HDMR model with thousands of input variables is computationally intensive. Therefore, as remarked earlier, ${\hat{S}}_{i}$ may be used first for feature pre-selection when the number of features is large, then followed by SCSA for refined feature selection, prioritization and correlation identification. If the number of features is not large, the feature pre-selection by ${\hat{S}}_{i}$ may be avoided.

#### svr-based HDMR algorithm with independent input variables

The function *f*(**x**) is approximated by SVR as [21]

$$\begin{array}{ccc} \hat{f}\left(x\right) & = & \left\langle w,\Phi \left(x\right)\right\rangle +b=\sum_{s=1}^{N}\left(\alpha_{s}-\alpha_{s}^{*}\right)\left\langle \Phi \left(x^{\left(s\right)}\right),\Phi \left(x\right)\right\rangle +b\\ & = & \sum_{s=1}^{N}\left(\alpha_{s}-\alpha_{s}^{*}\right)K\left(x^{\left(s\right)},x\right)+b, \end{array}$$ (16)

where *K*(**x**^(*s*)^, **x**) is referred to as a kernel. When an *HDMR kernel*, i.e., an HDMR expansion of kernels with different numbers of variables [20]

$$\begin{array}{ccc} K(x^{\left(s\right)},x) & = & c+\sum_{i=1}^{n}K_{i}\left(x_{i}^{\left(s\right)},x_{i}\right)+\sum_{1\leq i<j\leq n}K_{i}\left(x_{i}^{\left(s\right)},x_{i}\right)K_{j}\left(x_{j}^{\left(s\right)},x_{j}\right)+...\\ & + & \prod_{i=1}^{n}K_{i}\left(x_{i}^{\left(s\right)},x_{i}\right), \end{array}$$ (17)

where *c* ≥ 0, and the kernels $K_{i}\left(x_{i}^{\left(s\right)},x_{i}\right)$ satisfy the *zero expectation and mutual orthogonality conditions*

$$\begin{array}{c} E_{x_{i}}\left[K_{i}\left(x_{i}^{\left(s\right)},x_{i}\right)\right]=0,\;E_{x_{i},x_{j}}\left[K_{i}\left(x_{i}^{\left(s\right)},x_{i}\right)K_{j}\left(x_{j}^{\left(r\right)},x_{j}\right)\right]=0, \end{array}$$

is used in Eq (16), the svr-based HDMR expansion is obtained

$$\begin{array}{ccc} \hat{f}\left(x\right) & = & b+\sum_{s=1}^{N}\left(\alpha_{s}-\alpha_{s}^{*}\right)c+\sum_{i=1}^{n}[\sum_{s=1}^{N}\left(\alpha_{s}-\alpha_{s}^{*}\right)K_{i}\left(x_{i}^{\left(s\right)},x_{i}\right)]\\ & + & \sum_{1\leq i<j\leq n}[\sum_{s=1}^{N}\left(\alpha_{s}-\alpha_{s}^{*}\right)K_{i}\left(x_{i}^{\left(s\right)},x_{i}\right)K_{j}\left(x_{j}^{\left(s\right)},x_{j}\right)]+...\\ & + & \sum_{s=1}^{N}\left(\alpha_{s}-\alpha_{s}^{*}\right)\prod_{i=1}^{n}K_{i}\left(x_{i}^{\left(s\right)},x_{i}\right)\\ & = & f_{0}+\sum_{i=1}^{n}f_{i}\left(x_{i}\right)+\sum_{1\leq i<j\leq n}f_{ij}\left(x_{i},x_{j}\right)+...+f_{12..n}\left(x\right). \end{array}$$ (18)

This result shows that all non-constant HDMR component functions are represented as linear combinations of one variable kernels or products of some one variable kernels with combination coefficients *α*_*s*_ and $\alpha_{s}^{*}$. Thus, an HDMR model can be constructed by using an SVR algorithm, i.e., the determination of the unknown parameters *α*_*s*_ and $\alpha_{s}^{*}$, which is more efficient when fewer samples are adequate. This method is referred to as the svr-based HDMR algorithm. The *key* step is the construction of HDMR kernels satisfying the zero expectation and mutual orthogonality conditions. Various (polynomial, radial basis, exponential, Fourier) analytical HDMR kernels for independent variables with a known probability distribution of the variables have been constructed [20].

#### Extension of svr-based HDMR to correlated input variables

In most realistic experimental and clinical circumstances, the variables are correlated. For example, the occurrence of one causative metabolite is often accompanied by the occurrence of other causative metabolites. Moreover, the input variable probability distribution for experimental and clinical data is often explicitly unknown, and hence the analytical HDMR kernels cannot be constructed. Therefore, we need to extend the svr-based HDMR algorithm with *independent* input variables to *correlated* input variables without requiring knowledge of the input variable probability distribution. For the first order svr-based HDMR with correlated variables we only need to construct single variate HDMR kernels without requiring the knowledge of the pdf of input variables.

The HDMR kernel satisfies the property: zero expectation and its general formula is given by [28]

$$\begin{array}{c} K_{0}\left(x_{i},x_{i}^{\prime }\right)=K\left(x_{i},x_{i}^{\prime }\right)-\frac{\int K\left(x_{i},v\right)p_{i}\left(v\right)dv\int K\left(u,x_{i}^{\prime }\right)p_{i}\left(u\right)du}{\int \int K\left(u,v\right)p_{i}\left(u\right)p_{i}\left(v\right)dudv}. \end{array}$$ (19)

To construct HDMR kernels, we need to know the pdf, *p*_*i*_(*x*_*i*_)’s, for determination of the integrals in Eq (19). For real data, the pdf is likely correlated and unknown, but *implicitly* involved in the sampled data **x**^(*s*)^(*s* = 1, 2, …, *N*) because the samples are drawn according to their probability distribution function. Thus, without explicit knowledge of the pdf, we can construct the one-variate HDMR kernel numerically. Hence, the integrals in Eq (19) can be approximately computed by using Monte Carlo integration as follows.

The one-variate kernel $k(x_{i},x_{i}^{\prime })$ at *N* sample points $x_{i}^{\left(s\right)}\;\left(s=1,2,...,N\right)$, drawn from an explicitly unknown pdf, can be written as a matrix

$$\left[\begin{array}{cccc} k\left(x_{i}^{\left(1\right)},{\left(x_{i}^{\prime }\right)}^{\left(1\right)}\right) & k\left(x_{i}^{\left(1\right)},{\left(x_{i}^{\prime }\right)}^{\left(2\right)}\right) & \cdots & k\left(x_{i}^{\left(1\right)},{\left(x_{i}^{\prime }\right)}^{\left(N\right)}\right)\\ k\left(x_{i}^{\left(2\right)},{\left(x_{i}^{\prime }\right)}^{\left(1\right)}\right) & k\left(x_{i}^{\left(2\right)},{\left(x_{i}^{\prime }\right)}^{\left(2\right)}\right) & \cdots & k\left(x_{i}^{\left(2\right)},{\left(x_{i}^{\prime }\right)}^{\left(N\right)}\right)\\ \vdots & \vdots & \ddots & \vdots \\ k\left(x_{i}^{\left(N\right)},{\left(x_{i}^{\prime }\right)}^{\left(1\right)}\right) & k\left(x_{i}^{\left(N\right)},{\left(x_{i}^{\prime }\right)}^{\left(2\right)}\right) & \cdots & k\left(x_{i}^{\left(N\right)},{\left(x_{i}^{\prime }\right)}^{\left(N\right)}\right) \end{array}\right].$$ (20)

According to Monte Carlo integration, the first integral ∫*K*(*x*_*i*_, *v*)*p*_*i*_(*v*)*dv* at $x_{i}=x_{i}^{\left(k\right)}$ in the numerator in Eq (19) can be approximated as the average value of all the elements of the *k*th row in the above matrix. In the same fashion, the average value of all the elements of the *k*th column of the matrix is an approximation to the second integral $\int K\left(u,x_{i}^{\prime }\right)p_{i}\left(u\right)du$ at $x_{i}^{\prime }={\left(x_{i}^{\prime }\right)}^{\left(k\right)}$ in the numerator. The integral in the denominator of Eq (19) can be approximated as the average value of all the elements in the matrix.

As the above matrix is symmetric, the average values of all rows are just the average values of all columns. Therefore, the one-variate HDMR kernels can be constructed numerically from the *N* average values of the rows and the average value of all elements of the matrix *no matter whether the variables are independent or correlated and regardless of the pdf they possess*. For an arbitrary *x*_*i*_, the integrals in the numerator can be obtained by interpolation from the *N* average values for the $x_{i}^{\left(s\right)}$’s.

Using the one-variate HDMR kernels with correlated input variables, the first order HDMR expansion with correlated variables can be constructed by determining the parameters *α* and α* with the SVR algorithm. The SCSA indexes are then computed from the resultant HDMR component functions, and used for sensitivity analysis.

#### The procedure of the two stage method

The procedure for feature selection, prioritization and correlation identification for ASD-NEU classification by the new proposed two stage method is as follows:

- First, construct an SVM model with a properly chosen kernel (in the present ASD-NEU data, a linear kernel was found to be satisfactory) using all (24) metabolites as the input variables **x** whose values are normalized to [−1, 1], and the output *y* takes the values −1 and 1 to represent NEU and ASD, respectively. Then, collect the SVM scores for all 159 samples (76 normal children and 83 patients).
- For the present ASD-NEU data, the total number of 24 metabolites is not large, and one can directly use SCSA without feature pre-selection by ${\hat{S}}_{i}$. However, for illustration of the general two stage method we will still use ${\hat{S}}_{i}$ pre-selection first to remove some of the most unimportant metabolites defined as those with the smallest ${\hat{S}}_{i}$ values. Then, the first order svr-based HDMR expansion with all retained *x*_*i*_’s is constructed, and the corresponding first order SCSA indexes $S_{i}^{a},S_{i}^{b},S_{i}$ are computed to perform a final refined stage of feature selection, prioritization, and correlation identification.
- The selection of significant metabolites is performed by *a bottom-up method*, i.e., removing the most insignificant metabolites first with the smallest values of ${\hat{S}}_{i}$ and then with the smallest values of $S_{i}^{a}$, step-by-step, to obtain the final significant metabolites. There is no strict threshold for the magnitude of ${\hat{S}}_{i}$ or $S_{i}^{a}$ to remove insignificant metabolites. The *guiding rule* is that removal of metabolites does not significantly reduce the classification accuracy of the new SVM model with the retained metabolites.
  The reason for using the bottom-up method is that the importance order of metabolites obtained from either ${\hat{S}}_{i}$ or $S_{i}^{a}$ depends on SVM scores, which is a function of *all* the metabolites used in the SVM construction. We found that the order of the *top* significant metabolites is generally contaminated by including less-informative metabolites, and the result is not the same as their true order when only significant metabolites are used in SVM model construction. However, even if less-informative metabolites are included in SVM model construction, we may still reasonably assume that the metabolites with the smallest values of ${\hat{S}}_{i}$ or $S_{i}^{a}$ are the most insignificant and can be removed. As mentioned above, the smallest ${\hat{S}}_{i}$ does not necessarily mean that *x*_*i*_ is the most insignificant metabolite if the smallest value of ${\hat{S}}_{i}$ is obtained due to the negative correlations of *x*_*i*_ with other *x*_*j*_’s. However, in this case, removing *x*_*i*_ may result a significant reduction of classification accuracy of the new SVM model. If so, we keep *x*_*i*_ and remove the *x*_*j*_ with the next smallest value of ${\hat{S}}_{j}$ right above the ${\hat{S}}_{i}$.
- After removing some identified possibly insignificant metabolites, a new SVM model is constructed with the remaining metabolites, and the new SVM scores are collected to perform the above procedure again for further metabolite removal. The process continues until no more metabolite can be removed without significant reduction in accuracy of the next SVM model. The removal order of the removed metabolites is the inverse of their importance order, i.e., the earliest removed metabolites are deemed the least significant. Thus, using the inverse of the removal order of metabolites, the importance order of removed metabolites can be obtained.
- The final remaining metabolites are the most significant, and the values of $S_{i}^{a}$ give their importance order based on their independent contributions. The index $S_{i}^{b}$ provides the correlative contribution of *x*_*i*_ and all other variables *x*_*j*_’s correlated with *x*_*i*_, and *S*^*b*^(*ij*) gives the pairwise correlation between *x*_*i*_ and *x*_*j*_. The sum of *S*_*i*_ indicates the reliability of the overall sensitivity analysis. The first order HDMR component function *f*_*i*_(*x*_*i*_) plotted versus *x*_*i*_ provides the influence pattern of *x*_*i*_ to the predisposition of ASD.

## Results

### Feature pre-selection utilizing the main effect ${\hat{S}}_{i}$

The total number of 24 metabolites is not large, and we could directly perform SCSA. However, we still use the main effect ${\hat{S}}_{i}$ to remove the most unimportant metabolites first as an illustration of a general two stage procedure, especially valuable in situations having large numbers of input variables.

**Fig 4:**
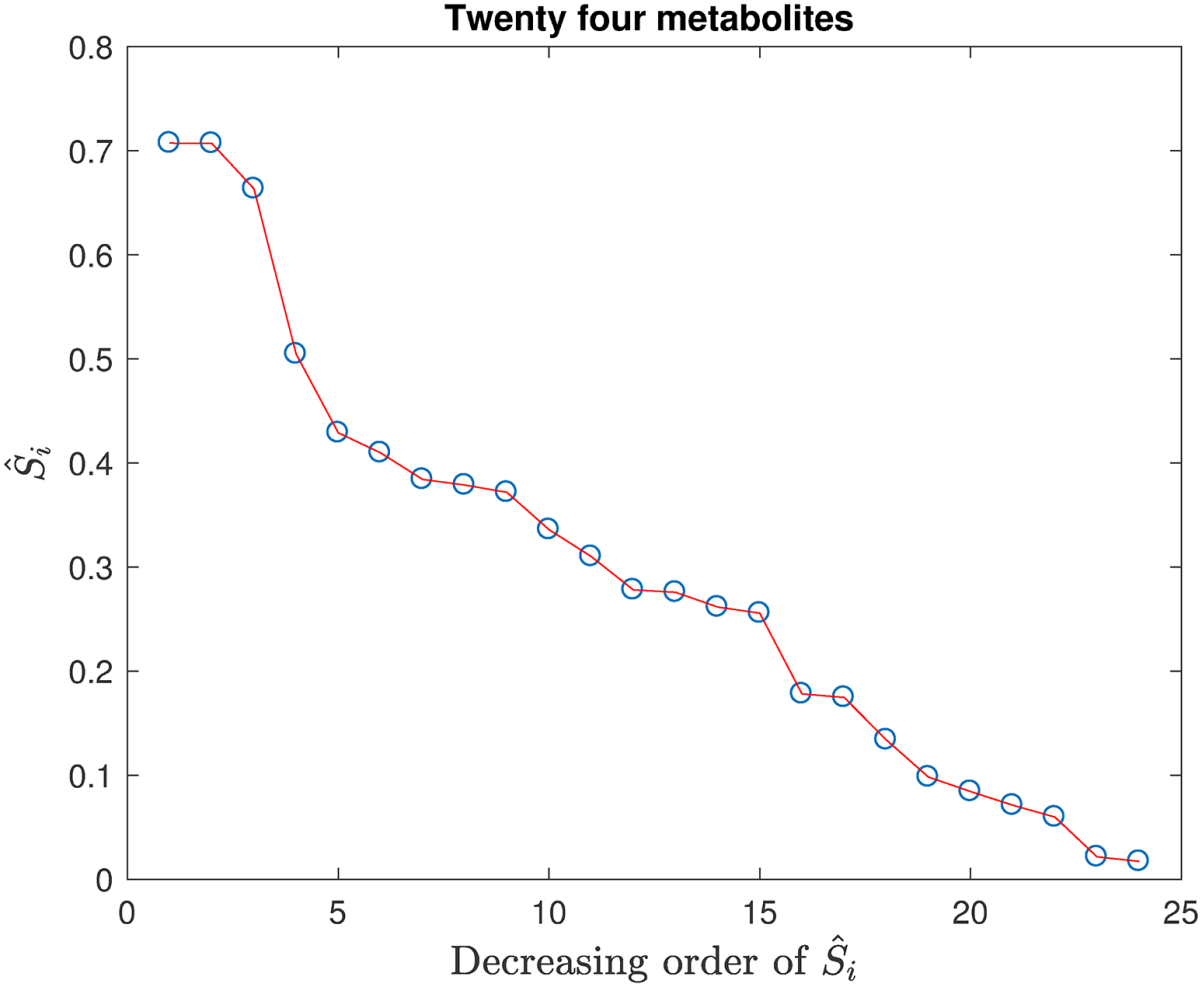
The 24 ${\hat{S}}_{i}$’s arranged in decreasing order of their magnitudes.

**Fig 5:**
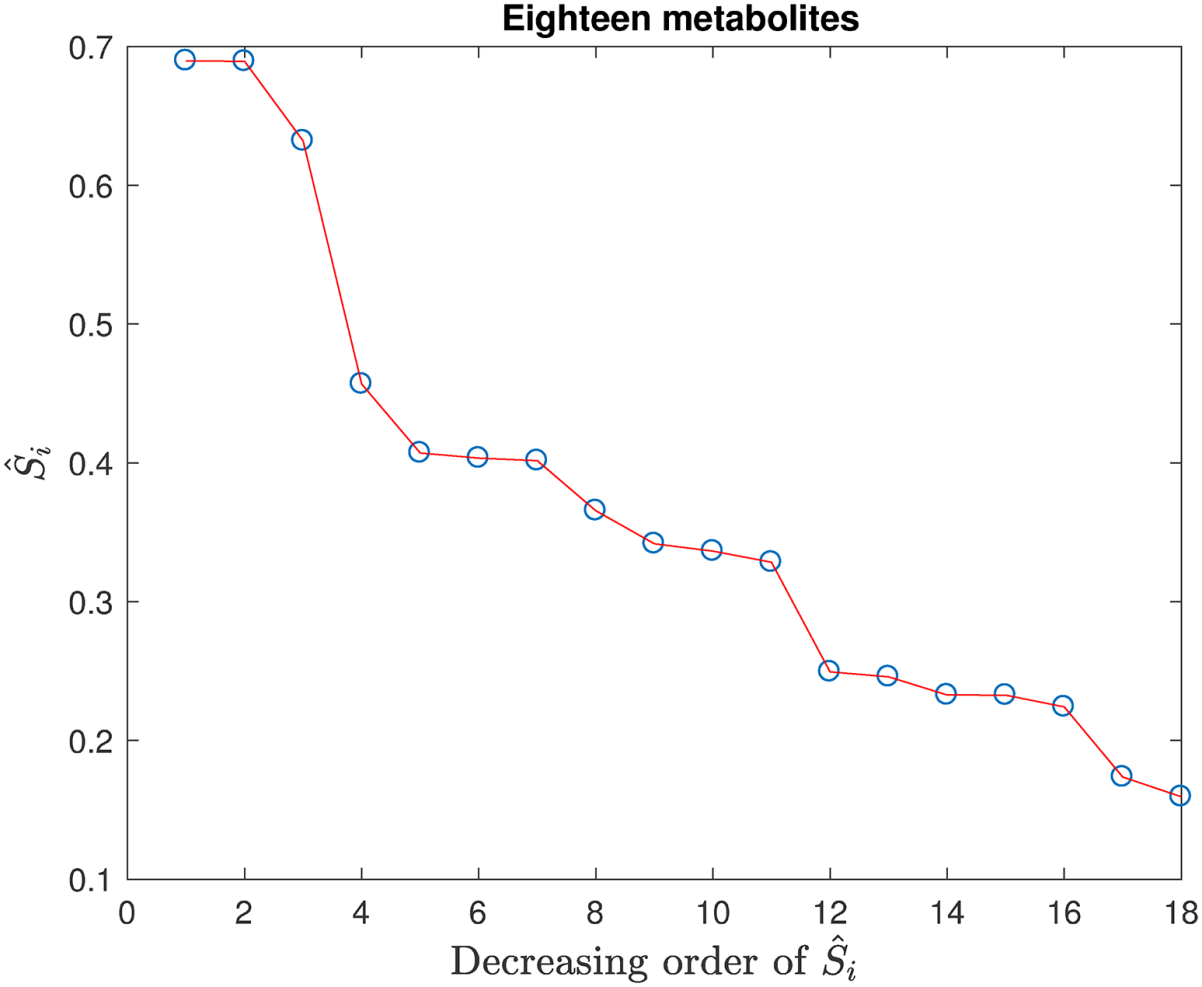
The ${\hat{S}}_{i}$’s obtained from the SVM model with 18 metabolites arranged in decreasing order of their magnitudes.

**Fig 6:**
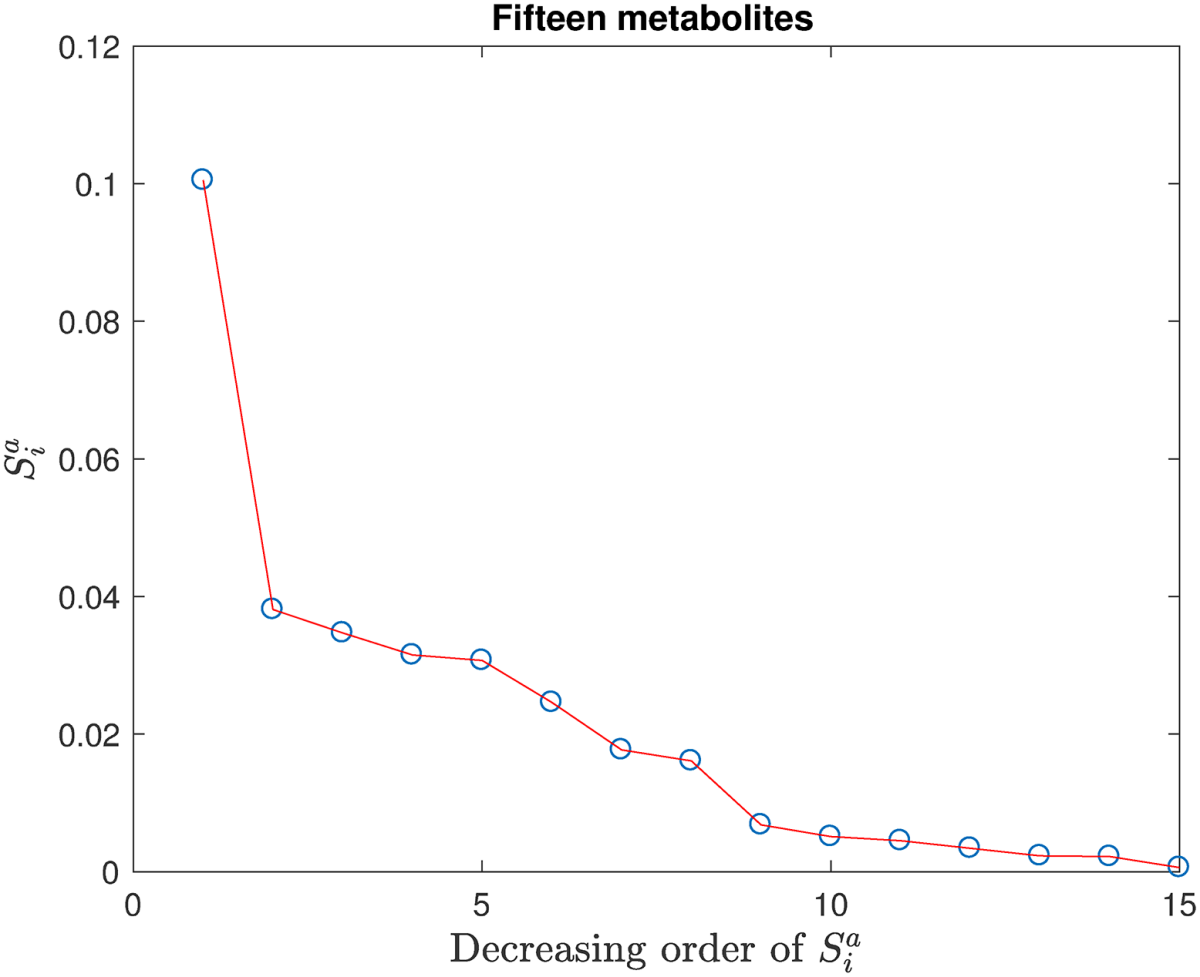
The 15 $S_{i}^{a}$’s arranged in decreasing order of their magnitudes.

Fig 4 gives the ${\hat{S}}_{i}$’s determined from the scores of the SVM model constructed by 10-fold cross-validation with the 24 metabolites. They are arranged in decreasing order of the magnitudes of ${\hat{S}}_{i}$. Note that the abscissa indexes in Figs 4–6 do not correspond to the sequentially labeled metabolites in Table 1; the particular metabolites of interest in these figures will be specified in the text discussion, as called for.

From Fig 4, we consider that the last six (going from 19 to 24) values of ${\hat{S}}_{i}$ are small, and their corresponding metabolites in Table 1 are *x*_7_ (Adenosine), *x*_11_ (Cys.-Gly.), *x*_19_ (Tyrosine), *x*_22_ (fCysteine), *x*_8_ (Homocysteine) and *x*_20_ (Tryptophan), respectively, which can be treated as the most unimportant and removed. After removing the latter six metabolites, a new SVM model was constructed with the retained 18 metabolites. The prediction accuracy of the new SVM model did not change significantly. Therefore, the removal of the last six metabolites in Fig 4 is deemed proper. The scores of the SVM model with the retained 18 metabolites were used to compute the new 18 main effect ${\hat{S}}_{i}$’s. The new ${\hat{S}}_{i}$’s arranged in decreasing order are given in Fig 5.

Fig 5 shows that the last two (the 17th and 18th) ${\hat{S}}_{i}$’s have the smallest magnitudes and their corresponding metabolites: *x*_3_ (SAH), *x*_21_ (fCystine) from Table 1 may be removed. We tested the removal of more, i.e., removing the last 3 or 4 metabolites (i.e., starting from the 15th or 16th position to the end in ${\hat{S}}_{i}$ sequence), but removing *x*_5_ (% DNA methylation) in the 15th position caused a significant reduction of accuracy of the new SVM model, so only the last three metabolites (*x*_2_ (SAM), *x*_3_ (SAH), *x*_21_ (fCystine)) were removed, resulting in a new subset of 15 metabolites. This overall procedure illustrates the means for systematic pre-selection of the likely significant metabolites utilizing ${\hat{S}}_{i}$.

### Feature selection and prioritization by the SCSA index $S_{i}^{a}$

We transferred from the ${\hat{S}}_{i}$-based feature pre-selection procedure to now use $S_{i}^{a},S_{i}^{b},S_{i}$ for more refined feature selection, prioritization and correlation identification. Using the score of the SVM model as the output *y* and the 15 metabolites pre-selected by ${\hat{S}}_{i}$ as the input variables, the first order HDMR expansion was constructed by the svr-based HDMR algorithm with correlated variables, and the corresponding first order SCSA indexes $S_{i}^{a},S_{i}^{b},S_{i}$ were computed. The 15 $S_{i}^{a}$’s are arranged in decreasing order of their magnitudes in Fig 6.

From Fig 6, we see that the last few $S_{i}^{a}$’s are very small suggesting that their corresponding metabolites may be removed. Removal of metabolites should be carefully performed such that the accuracy of the new SVM model with the retained metabolites is not significantly influenced. In this way, significant metabolites will not be *mistakenly* removed. A reliable procedure is removal of one metabolite at a time, but it may not be efficient when the number of metabolites is large. In the present case with just 15 metabolites, we did remove insignificant metabolites one-by-one. Thus, in seven separate sequential steps, *x*_14_ (GSSG), *x*_13_ (fGSH), *x*_12_ (tGSH), *x*_15_ (fGSH/GSSG), *x*_4_ (SAM/SAH), *x*_9_ (Cysteine) and *x*_1_ (Methionine) were removed to obtain a set of 8 metabolites. For brevity, the detailed results of the seven steps are not given here. Only the SCSA indexes arranged in decreasing order of $S_{i}^{a}$ for the *subsequent* few steps in going from 8 to 6 remaining metabolites are given below in Tables 3–5.

**Table 3:** The $S_{i}^{a},S_{i}^{b},S_{i}$ obtained from the first order HDMR expansion with 8 metabolites.

Order	Variable	$S_{i}^{a}$	$S_{i}^{b}$	*S*_*i*_	Metabolite
1	*x*_6_	0.0623	0.0962	0.1585	8-OHG
2	*x*_17_	0.0518	0.0823	0.1340	Chlorotyrosine
3	*x*_5_	0.0493	0.0604	0.1098	% DNA methylation
4	*x*_24_	0.0428	0.1133	0.1561	% oxidized glutathione
5	*x*_16_	0.0401	0.1081	0.1582	tGSH/GSSG
6	*x*_23_	0.0351	0.0547	0.0899	fCystine/fCysteine
7	*x*_10_	0.0343	0.0524	0.0867	Glu.-Cys.
8	*x*_18_	0.0266	0.0772	0.1039	Nitrotyrosine
Sum		0.3424	0.6447	0.9871	

**Table 4:** The $S_{i}^{a},S_{i}^{b},S_{i}$ obtained from the first order HDMR expansion with 7 metabolites.

Order	Variable	$S_{i}^{a}$	$S_{i}^{b}$	*S*_*i*_	Metabolite
1	*x*_5_	0.1122	0.0653	0.1774	% DNA methylation
2	*x*_10_	0.0835	0.0738	0.1571	Glu.-Cys.
3	*x*_17_	0.0757	0.1032	0.1790	Chlorotyrosine
4	*x*_23_	0.0561	0.0531	0.1091	fCystine/fCysteine
5	*x*_24_	0.0479	0.1090	0.1568	% oxidized glutathione
6	*x*_6_	0.0382	0.0751	0.1134	8-OHG
7	*x*_16_	0.0201	0.0789	0.0990	tGSH/GSSG
Sum		0.4337	0.5583	0.9920	

**Table 5:** The $S_{i}^{a},S_{i}^{b},S_{i}$ obtained from the first order HDMR expansion with 6 metabolites.

Order	Variable	$S_{i}^{a}$	$S_{i}^{b}$	*S*_*i*_	Metabolite
1	*x*_5_	0.1137	0.0668	0.1805	% DNA methylation
2	*x*_24_	0.0926	0.1224	0.2149	% oxidized glutathione
3	*x*_10_	0.0875	0.0708	0.1583	Glu.-Cys.
4	*x*_17_	0.0800	0.1027	0.1827	Chlorotyrosine
5	*x*_23_	0.0755	0.0604	0.1359	fCystine/fCysteine
6	*x*_6_	0.0427	0.0774	0.1207	8-OHG
Sum		0.4920	0.5004	0.9924	

From Tables 3–5, we see that *x*_18_ (Nitrotyrosine) and *x*_16_ (tGSH/GSSG) were removed sequentially. The importance order of the six metabolites in Table 5 is different from that in Tables 3 and 4. As discussed before, the order of the top significant metabolites is likely contaminated by the less-informative metabolites included in the SVM model construction; the true order is only obtained when the actually significant metabolites are used in SVM model construction. This is why the bottom-up method is utilized to correctly obtain the most important metabolites.

According to the values of the $S_{i}^{a}$’s given in Table 5, *x*_6_ (8-OHG) could be considered as another candidate for removal. Upon doing so, the accuracy of SVM model with the first 5 metabolites in Table 5 significantly decreased. The prediction error of the SVM model is represented by the mean classification error (MCE), the misclassifications for both ASD and NEU.

$$\begin{array}{c} \text{MCE}=\frac{\text{Number}\;\text{of}\;\text{misclassifications}}{\text{total}\;\text{number}\;\text{of}\;\text{data}}. \end{array}$$

The MCE’s of the SVM models with 4 to 8 metabolites obtained from all 159 samples with 10-fold cross-validation are given in Table 6, which shows that a significant increase of SVM prediction error occurs starting from 5 metabolites. This implies that *x*_6_ (8-OHG) is still a causative metabolite for ASD and we have conservatively retained it. Thus, the six metabolites in Table 5 are chosen as the *final* significant metabolites. As explained earlier, their importance order defined by their independent contribution is determined by their $S_{i}^{a}$ values.

**Table 6:** The MCE’s of SVM models with 4 to 8 metabolites.

Number of metabolites	8	7	6	5	4
MCE	0.0126	0.0126	0.0126	0.0314	0.0626

As remarked before, the bottom-up method of feature removal gives the importance order inversely for all removed metabolites at each step. Thus, combining the importance order for the identified six significant metabolites given in Table 5 with the importance order of *all* removed metabolites, we obtain the overall prioritization order for all the 24 metabolites in Table 7.

**Table 7:** The prioritization order of FOCM/TS metabolites.

Order	Variable	Metabolite	Order	Variable	Metabolite
1	*x*_5_	% DNA methylation	13	*x*_12_	tGSH
2	*x*_24_	% oxidized glutathione	14	*x*_13_	fGSH
3	*x*_10_	Glu.-Cys.	15	*x*_14_	GSSG
4	*x*_17_	Chlorotyrosine	16	*x*_2_	SAM
5	*x*_23_	fCystine/fCysteine	17	*x*_3_	SAH
6	*x*_6_	8-OHG	18	*x*_21_	fCystine
7	*x*_16_	tGSH/GSSG	19	*x*_7_	Adenosine
8	*x*_18_	Nitrotyrosine	20	*x*_11_	Cys.-Gly.
9	*x*_1_	Methionine	21	*x*_19_	Tyrosine
10	*x*_9_	Cysteine	22	*x*_22_	fCysteine
11	*x*_4_	SAM/SAH	23	*x*_8_	Homocysteine
12	*x*_15_	fGSH/GSSG	24	*x*_20_	Tryptophan

The metabolites close to the end of this prioritization sequence are the least informative to ASD. If ${\hat{S}}_{i}$ were not used for feature pre-selection, and only $S_{i}^{a}$ were employed, then the prioritization order of the metabolites at the last several positions might be different, but this difference is unimportant because these metabolites are all insignificant no matter what order they have.

The differences between the $S_{i}^{a}$ values for the most important 6 metabolites given in Table 5 are not very large, especially, for the middle 4 metabolites where the difference of neighbor metabolites is only ∼0.01. Such a small difference may be real or caused by the small sample size (159 samples), experimental errors (measurements of the metabolites) and numerical errors (construction of the SVM and HDMR models). Therefore, their order may not be very significant, and possibly some of them are almost equally important.

Ignoring the precise orderings, the resulting best 7 and 6 metabolite subsets from Table 7 are exactly the same as those given in Table 2 reported by Howsmon et al. [8] However, to obtain these optimal sets of metabolites Howsmon et al. performed an enormous number of Fisher discrimination classification tests, but we obtained the same results with only a few steps.

Note that the first 5 metabolites in the sequence given in Table 7 is different from that given by Howsmon et al. As shown below, our selection of the most important 5 metabolites provides better accuracy in ASD classification than that selected by Howsmon et al. This result implies that the new method more reliably identifies the significant metabolites.

To test the validity of our selections, the prediction error of the SVM models with the best combination of *k*(= 5–8) metabolites chosen by the first *k* metabolites in Table 7 was computed. For comparison, the prediction error of the SVM model with the 5 metabolites chosen by Howsmon et al. is also included.

To determine the prediction error, Howsmon et al. used leave-one-out cross-validation [8]. However, the leave-one-out algorithm may be a good method to construct a model with a limited amount of data, but may not be the *best* algorithm to estimate the prediction error because every datum is still involved in the model construction anyway. So we use a different method. All 159 data points are *randomly* separated into training (100 points) and testing (59 points) data subsets. The SVM model with a linear kernel for a given subset of metabolites was constructed *only* from the training data with 10-fold cross-validation, which was then used to predict the testing data not involved in the model construction. We ran these tests 100 times and calculated the mean and standard deviation of the 100 MCE’s for the training and testing data of SVM models with different numbers of metabolites. The results are given in Table 8.

**Table 8:** The mean and standard deviation of MCE for training and testing data.

Number of metabolites	Metabolites	Training data		Testing data	
		Mean	Std	Mean	Std
5*	*x*_5_, *x*_6_, *x*_10_, *x*_23_, *x*_24_	0.0262	0.0478	0.0537	0.0492
5	*x*_5_, *x*_10_, *x*_17_, *x*_23_, *x*_24_	0.0266	0.0155	0.0437	0.0226
6	*x*_5_, *x*_6_, *x*_10_, *x*_17_, *x*_23_, *x*_24_	0.0135	0.0114	0.0392	0.0268
7	*x*_5_, *x*_6_, *x*_10_, *x*_16_, *x*_17_, *x*_23_, *x*_24_	0.0147	0.0118	0.0356	0.0257
8	*x*_5_, *x*_6_, *x*_10_, *x*_16_, *x*_17_, *x*_18_, *x*_23_, *x*_24_	0.0124	0.0010	0.0313	0.0238

*selected by Howsmon et al.

In Table 8, the mean MCE is less than 2% and 4% for training and testing data (corresponding to an average of 2 and 2.36 misclassifications) respectively when the SVM model contains 6 and more metabolites. Therefore, the prediction accuracy is larger than 98% and 96% for training and testing data respectively, which are satisfactory. The results in Table 8 show that the MCE decreases when the number of metabolites increases. The MCE’s for the 5 metabolites selected by our method and Howsmon et al. are very close, but our selection has a little better accuracy.

### Correlation identification by the SCSA indexes $S_{i}^{b}$ and *S*^*b*^(*ij*)

Correlation of metabolites means *simultaneous variations* or *variation dependency* of metabolites, which may be caused by different sources such as correlated metabolites are on the same path leading to predisposition of ASD; correlated metabolites can have either a promotion or inhibition effect on each other. The doctors or researchers working on ASD may find correct interpretation of these correlations. Discovery of correlations is important for a pathological interpretation and comprehensive treatment of ASD.

The matrix *S*^*b*^(*ij*)(*i*, *j* = 1, 2, …, *n*) gives all the information of metabolite pairwise correlation (see Eq (13)). The *S*^*b*^(*ij*) matrix for the 6 important metabolites is given below where the metabolite order is *x*_5_, *x*_24_, *x*_10_, *x*_17_, *x*_23_, *x*_6_ given in Table 5,

$$\begin{array}{c} {}[\begin{array}{cccccc} 0.1137 & 0.0220 & -0.0066 & 0.0141 & 0.0190 & 0.0183\\ 0.0220 & 0.0926 & 0.0343 & 0.0237 & 0.0224 & 0.0199\\ -0.0066 & 0.0343 & 0.0875 & 0.0386 & -0.0045 & 0.0089\\ 0.0141 & 0.0237 & 0.0386 & 0.0800 & 0.0098 & 0.0166\\ 0.0190 & 0.0224 & -0.0045 & 0.0098 & 0.0755 & 0.0136\\ 0.0183 & 0.0199 & 0.0089 & 0.0166 & 0.0136 & 0.0427 \end{array}]. \end{array}$$ (21)

Note that the diagonal elements are $S_{i}^{a}$’s; and the sum of all elements except the diagonal one in the *i*th row (or column) of the *S*^*b*^(*ij*) matrix is just $S_{i}^{b}$, i.e., the sum of all pairwise correlative contributions of *x*_*i*_ with all other *x*_*j*_’s; and the sum of all elements in the *i*th row (or column) of the *S*^*b*^(*ij*) matrix is *S*_*i*_.

Table 5 shows that (i) the magnitudes of all six $S_{i}^{b}$ are comparable to the magnitudes of corresponding $S_{i}^{a}$, and (ii) $\sum_{i}S_{i}^{b}\approx \sum_{i}S_{i}^{a}\approx 0.5$ (i.e., the total contribution of all six metabolites is composed of half independent and half correlated contributions). Hence, all six significant metabolites are strongly correlated to one another, and ASD predisposition is not caused or represented by *individual* but rather *synergistic* effects of the abnormality due to the six metabolites. Especially, *x*_24_ (% oxidized glutathione, row 2 in the *S*^*b*^(*ij*) matrix) and *x*_17_ (Chlorotyrosine, row 4 in the *S*^*b*^(*ij*) matrix) have the largest values 0.1224 and 0.1027 for $S_{i}^{b}$, respectively (see Table 5), showing that within the six metabolites, they are most correlated to other metabolites.

**Fig 7:**
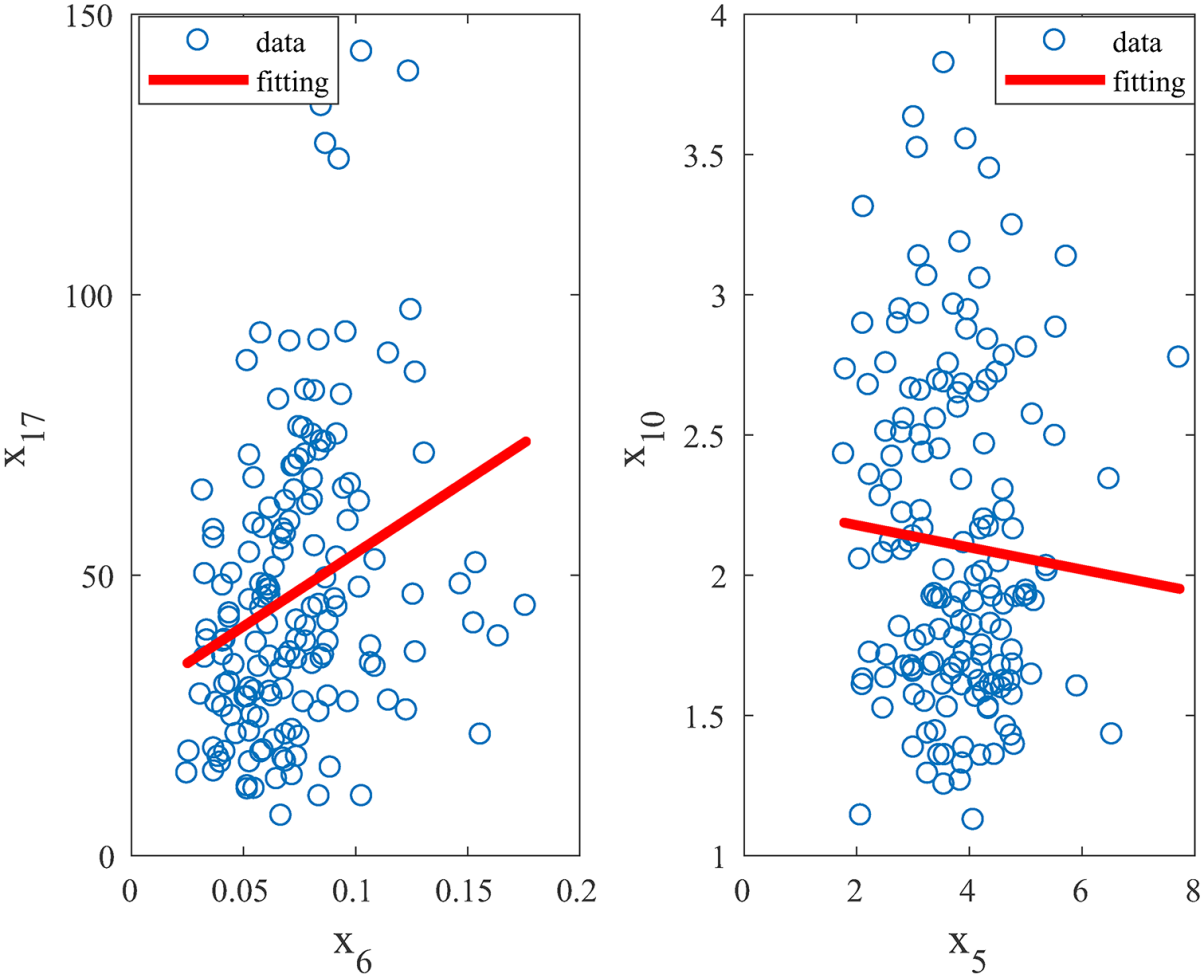
Relations between the measurements of *x*_17_ versus *x*_6_ and *x*_10_ versus *x*_5_.

The off-diagonal elements in the *S*^*b*^(*i*, *j*) matrix give the pairwise correlation between *x*_*i*_ and *x*_*j*_. Consider two pairwise correlations: the (6, 4)- and (1, 3)-entry of the *S*^*b*^(*ij*) matrix, i.e., *S*^*b*^(*x*_6_, *x*_17_) = 0.0166 and *S*^*b*^(*x*_5_, *x*_10_) = −0.0066. Fig 7 plots the relationship between the measurements of *x*_17_ versus *x*_6_ and *x*_10_ versus *x*_5_, along with the linear fitting of their correlations (since the linear kernel is used in the SVM modeling for the present ASD-NEU data, the correlation here is linear; in general case, the correlation may be non-linear).

When *x*_6_ increases, *x*_17_ has a tendency to increase while when *x*_5_ increases, *x*_10_ has a tendency to decrease consistent in both cases with the sign of their *S*^*b*^(*ij*)’s. Thus, the magnitudes and signs of *S*^*b*^(*x*_6_, *x*_17_) and *S*^*b*^(*x*_5_, *x*_10_), respectively, represent the correlative strength and nature of the respective pairs of metabolites. Similar interpretations can be made for other pairwise correlations. Within all the pairwise correlations, the largest are the (2, 3)- and (3, 4)-entries of the *S*^*b*^(*ij*) matrix, i.e., *S*^*b*^(*x*_24_, *x*_10_) = 0.0343 and *S*^*b*^(*x*_10_, *x*_17_) = 0.0386.

### Influence patterns of metabolites upon ASD predisposition

**Fig 8:**
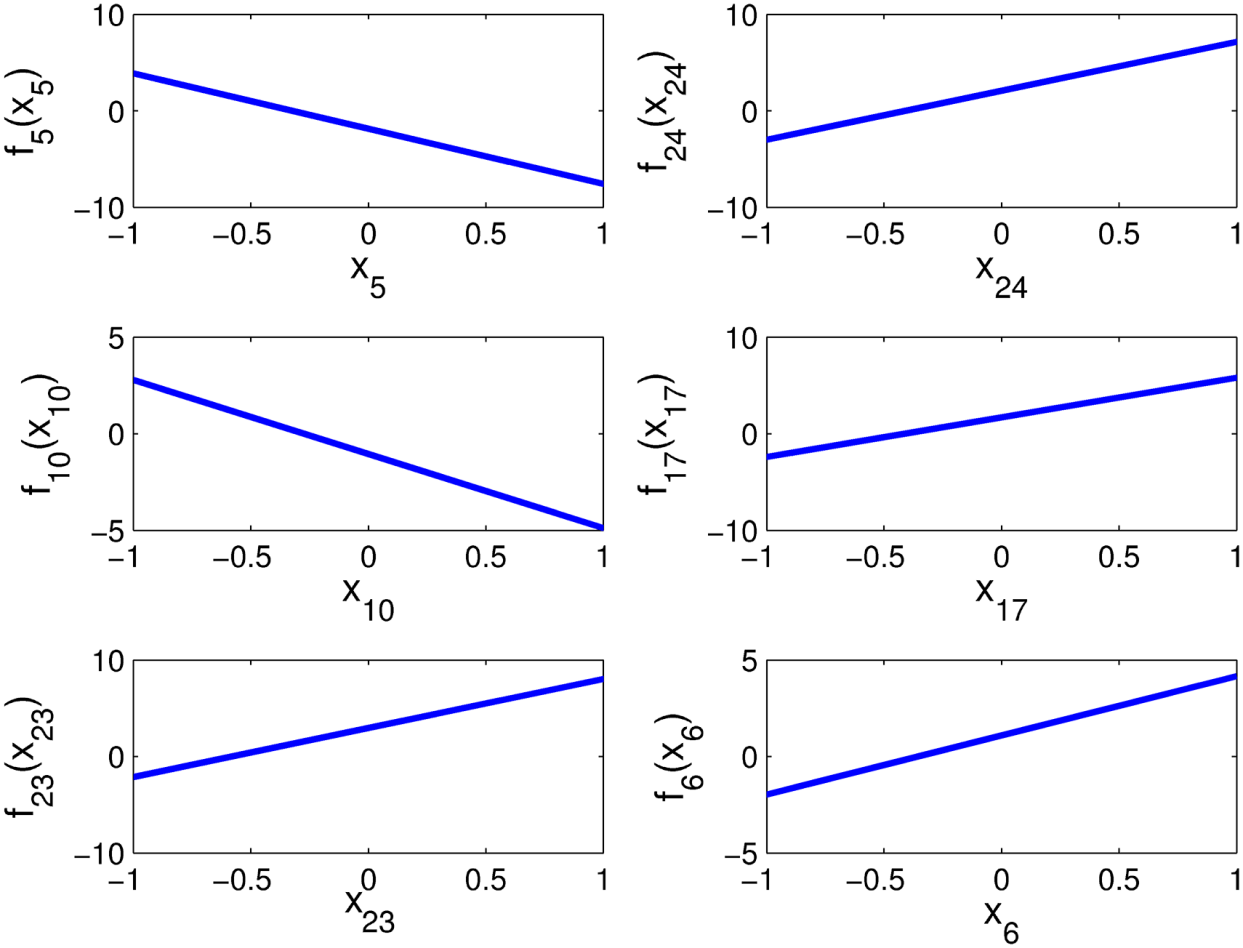
The first order component functions of the HDMR model with the six metabolites (all metabolites are normalized to [−1, 1]).

The first order HDMR component functions provide information about the influence pattern of metabolites upon ASD predisposition. Fig 8 gives the first order HDMR component functions for the HDMR model with the six metabolites.

As setting ASD = 1 and NEU = −1 in SVM classification, respectively, Fig 8 shows that larger values of *x*_6_ (8-OHG), *x*_17_ (Chlorotyrosine), *x*_23_ (fCystine/fCysteine) and *x*_24_ (% oxidized glutathione) but smaller values of *x*_5_ (% DNA methylation) and *x*_10_ (Glu.-Cys.) imply stronger ASD predisposition. Thus, the former metabolites might enhance, but the latter metabolites might inhibit ASD predisposition. This information is valuable for aiding a pathological interpretation of the influence of the metabolites on ASD.

## Conclusion

The discovery of the metabolite abnormalities in FOCM/TS pathways that have effects upon increasing or decreasing ASD predisposition is a significant advance in understanding ASD. The identification of which metabolites are relevant to ASD and how they either inhibit or enhance ASD is important to discern. In this paper, we present a new method that utilizes the scores produced in SVM modeling combined with HDMR sensitivity analysis to effectively and efficiently identify causative metabolites in FOCM/TS pathways, rank their importance, and discover their independent and correlative action patterns upon ASD. We expect that such information will not only be important for a pathological interpretation but also for early diagnosis and ideally providing a path leading to a comprehensive treatment of ASD. These prospects and analyses will most surely benefit from additional metabolite data, and this paper serves the purpose to provide an efficient means of extracting such information even with increasing numbers of metabolites for assessment. The new method, with only tens model runs, can identify the best combination of metabolites in FOCM/TS pathways leading to ASD, in comparison to a previous analysis method requiring hundreds of thousands model runs [8]. The same method introduced in the present paper may be useful for different types of biochemical applications and in other areas of data.

## Supporting information

S1 Dataset Biochemical and adaptive behavior data from ASD, NEU, and SIB participants. (CSV)
